# Simultaneous Saccharification and Fermentation of Sugar Beet Pulp for Efficient Bioethanol Production

**DOI:** 10.1155/2016/3154929

**Published:** 2016-09-19

**Authors:** Joanna Berłowska, Katarzyna Pielech-Przybylska, Maria Balcerek, Urszula Dziekońska-Kubczak, Piotr Patelski, Piotr Dziugan, Dorota Kręgiel

**Affiliations:** ^1^Department of Fermentation Technology, Institute of Fermentation Technology and Microbiology, Faculty of Biotechnology and Food Sciences, Lodz University of Technology, Wólczańska Str. 171/173, 90-924 Łódź, Poland; ^2^Department of Spirit and Yeast Technology, Institute of Fermentation Technology and Microbiology, Faculty of Biotechnology and Food Sciences, Lodz University of Technology, Wólczańska Str. 171/173, 90-924 Łódź, Poland; ^3^Department of Technical Microbiology, Institute of Fermentation Technology and Microbiology, Faculty of Biotechnology and Food Sciences, Lodz University of Technology, Wólczańska Str. 171/173, 90-924 Łódź, Poland

## Abstract

Sugar beet pulp, a byproduct of sugar beet processing, can be used as a feedstock in second-generation ethanol production. The objective of this study was to investigate the effects of pretreatment, of the dosage of cellulase and hemicellulase enzyme preparations used, and of aeration on the release of fermentable sugars and ethanol yield during simultaneous saccharification and fermentation (SSF) of sugar beet pulp-based worts. Pressure-thermal pretreatment was applied to sugar beet pulp suspended in 2% w/w sulphuric acid solution at a ratio providing 12% dry matter. Enzymatic hydrolysis was conducted using Viscozyme and Ultraflo Max (Novozymes) enzyme preparations (0.015–0.02 mL/g dry matter). Two yeast strains were used for fermentation: Ethanol Red (*S. cerevisiae*) (1 g/L) and* Pichia stipitis* (0.5 g/L), applied sequentially. The results show that efficient simultaneous saccharification and fermentation of sugar beet pulp was achieved. A 6 h interval for enzymatic activation between the application of enzyme preparations and inoculation with Ethanol Red further improved the fermentation performance, with the highest ethanol concentration reaching 26.9 ± 1.2 g/L and 86.5 ± 2.1% fermentation efficiency relative to the theoretical yield.

## 1. Introduction

Sugar beet is grown and processed in all countries of the European Union (with the exception of Luxembourg) and plays an important role in sustaining jobs and local economies in many rural areas. Some 300,000 farms are involved in sugar beet production and the sugar industry is also a large employer [1]. Byproducts from the processing of sugar beet include beet leaves and sugar beet pulp. One ton of sugar beet yields on average 160 kg of sugar, 500 kg of wet pulp, and 38 kg of molasses. The exhausted beet material, which remains after diffusion with hot water to draw the sugar from the beets, is called pulp. It is usually pressed and/or dried for animal feed [2]. Annual production of beet pulp in the EU amounts to around 8 million tons of pressed and 5.5 million tons of dried product [1].

Lignocellulosic biomass, including sugar beet pulp (SBP), is a promising carbon source for the production of bio-based fuels and chemicals. SBP can be converted into fuel ethanol through chemical and/or enzymatic hydrolysis and via biochemical pathways. SBP consists mainly of polysaccharides such as cellulose (22–30%), hemicelluloses (24–32%), lignin (1-2%), and pectin (38–62%), which constitute up to 75–85% of the dry matter [3–5]. Before fermentation, the cell-wall material must be degraded into fermentable monosaccharides [6]. Before enzymatic hydrolysis of cellulose and hemicellulose into fermentable monosaccharides, lignocellulosic feedstocks are often structurally modified by mild pretreatment. The goal is to break the lignin seal and disrupt the crystalline structure of the cellulose [6–8].

Cellulose and hemicellulose can be hydrolytically broken down into simple sugars by cellulases and hemicellulases, respectively, or by acids (e.g., sulfuric acid). Hexoses (glucose, galactose, and mannose) are fermented to ethanol by many naturally occurring microorganisms, but pentoses such as xylose and arabinose can be fermented to ethanol by relatively few native strains and usually at relatively low yields [9]. Xylose and arabinose generally comprise a significant fraction of agricultural residues and must be utilized to make biomass processing economically viable [10].

*Saccharomyces cerevisiae*, which remains the most widely used yeast for ethanol biosynthesis, produces ethanol by fermenting hexose sugars but is unable to ferment pentose sugars [11].* S. cerevisiae* is incapable of growing on xylose and does not produce ethanol, although it does produce limited amounts of xylitol. A possible reason for this may be cofactor limitation. Xylose transport is also less efficient than glucose transport. Xylose is transported four times more slowly than glucose under aerobic conditions and twice as slowly under anaerobic conditions [12]. Some yeasts, such as* Candida shehatae*,* Candida tropicalis*, and* Pichia stipitis*, can ferment xylose and hexoses with relatively high yields [13] but have low ethanol tolerances, and ethanol concentrations over 30–35 g/L inhibit their metabolisms [14, 15]. The ability of these yeasts to metabolize xylose depends on culture oxygenation [16, 17].

When enzymatic hydrolysis is performed independently of the fermentation step (known as separate hydrolysis and fermentation, SHF), this results in high concentrations of lower saccharides, which expose the yeast to osmotic stress and even cause substrate inhibition. By adding the yeast and the enzymes which catalyze the hydrolysis of polysaccharides at the same time, so that the two processes occur simultaneously (simultaneous saccharification and fermentation, SSF), this effect can be reduced, since fermentable sugars are released and consumed continuously throughout the process [18, 19].

A number of reports in the literature discuss ethanol production using lignocellulosic biomass via simultaneous saccharification and fermentation [20]. However, few describe the utilization of SBP. With this substrate, however, costly thermochemical pretreatment might be avoided, due to its low lignin and high pectin contents. The proposed solution requires only simple and mild pretreatment procedures. Zheng et al. [21] report successful conversion of SBP into ethanol by SSF using several genetically modified ethanologenic bacteria, including* Escherichia coli* KO11,* Klebsiella oxytoca* P2, and* Erwinia chrysanthemi* EC16. In our study, only nonmodified yeasts strains were used. We also plan to conduct the bioconversion process using mixed cultures (conventional and nonconventional yeast), to solve the problem of xylose consumption.

The objective of this study was to determine the effects of various pretreatment methods and varying dosages of cellulase and hemicellulase enzyme preparations on polysaccharide hydrolysis and simultaneous saccharification and fermentation (SSF) of sugar beet pulp using two yeast strains applied sequentially: Ethanol Red (*S. cerevisiae*), recommended for hexose sugar fermentation, and* Pichia stipites*, which has good xylose fermentation ability. The effect of fermentation medium aeration after inoculation with* Pichia stipitis* on the efficiency of the process was also evaluated.

## 2. Materials and Methods

### 2.1. Feedstock

Fresh sugar beet pulp (SBP) was obtained from the Dobrzelin Sugar Factory (Poland) and stored at −20°C until used.

### 2.2. Enzymes

SBP was hydrolyzed using the commercial enzyme preparations Viscozyme (a multienzyme complex containing a wide range of carbohydrases, including arabanase, cellulase, *β*-glucanase, hemicellulase, and xylanase) and Ultraflo Max (endo-1,3(4)-*β*-glucanase; endo-1,4-xylanase) (Novozymes A/S, Denmark). Enzyme preparations were applied simultaneously, at loading rates ranging from 0.01 to 0.07 mL/g dry matter. Individual sugar yields were compared to determine the most effective dose for C6 and C5 sugar liberation.

### 2.3. Yeast Strains, Media, and Cultivation Conditions

Fermentation was carried out using a preparation of Ethanol Red dry distillery yeast (*S. cerevisiae*) (Fermentis Division S.I. Lesaffre, France) and* Pichia stipitis* NCYC 1541 (National Collection of Yeast Cultures, UK), applied sequentially. The process was initiated using yeast Ethanol Red (1 g d.m./L) and after 24 h the worts were inoculated with* Pichia stipitis* (0.5 g/L).* Pichia stipitis* was subcultured at 30°C on a solid YPG medium containing 1% yeast extract, 2% peptone, 2% glucose, and 2% agar. Two-step propagation was performed to obtain the* P. stipitis* inoculum. In the first step, stationary stage, inoculum cultures were grown for 24 h at 30°C in 100 mL Erlenmeyer flasks filled with 50 mL of liquid YPG medium supplemented with xylose (1%). The inoculum obtained was then transferred under sterile conditions into 1 L flasks containing 100 mL of the YPG medium and xylose. Propagation was carried out in shaken cultures for 48 hours at 30°C. The biomass obtained was centrifuged, washed twice with sterile physiological saline, and centrifuged again. After suspending the biomass in saline, the biomass yield was determined by drying the sample to a constant weight at 105°C. The yeast slurry was added to the worts at a ratio of 0.5 g d.m. of yeast/L wort.

### 2.4. Preparation of Worts

To prepare the worts for fermentation, SBP was milled to obtain 0.8–1.0 mm particles and a portion of the pulp (100 g) diluted with fresh water or with 2% w/w sulfuric acid (192 mL), to obtain mixtures with dry matter content ca. 12% w/w. Next, the mixtures were subjected to two types of pretreatment: (1) thermal pretreatment in a lab-scale autoclave by heating the sugar beet pulp to 121°C for 30 or 60 min at 0.1 MPa and (2) ultrasound pretreatment with a SONOPULS HD 2200 homogenizer in continuous mode, set to 50% or 100% amplitude (ultrasound power 400 W, 24 kHz) for 20 min. The control samples were not subjected to either thermal or ultrasound pretreatment. After pretreatment, the worts were adjusted to pH 4.8 using 25% (w/w) sodium hydroxide before undergoing a process of simultaneous saccharification and fermentation, with or without the “activation” phase. Two variants of wort with “enzymatic activation” were prepared. In Variant I, the medium was digested with Viscozyme and Ultraflo Max enzyme preparations (each at a dose of 0.02 mL/g d.m.) and continually stirred and heated to 40°C for 6 h before inoculation with yeast. In Variant II, the medium was digested using the same preparations, each at doses of 0.015 mL/g d.m., stirred continuously, and heated to 48–50°C for 6 hours before inoculation with yeast. In Variant II, the worts were supplemented with (NH_4_)_2_HPO_4_ (0.3 g/L) and inoculated with yeast. In fermentation trials without previous enzymatic activation, the samples were digested with enzyme preparations (each at doses of 0.02 mL/g d.m.), supplemented with (NH_4_)_2_HPO_4_ (0.3 g/L), and immediately inoculated with yeast.

### 2.5. Fermentation

The fermentation experiments were carried out in 1 L glass flasks, each containing approximately 0.5 L of wort. Fermentation was initiated using 1 g of Ethanol Red distillery yeast (*S. cerevisiae*) per 1 L of wort. The yeast was first hydrated and acid-washed (15 min incubation of cells suspended in water with the addition of 25% w/w sulfuric acid solution, pH 2.5, at room temperature). The flasks were closed with stoppers equipped with fermentation pipes, filled with glycerol, and kept in a thermostat-regulated room at 37 ± 1°C. Fermentation was continued over 24 hours, at the end of which the specimens were inoculated with the* Pichia stipitis* yeast strain (0.5 g/L). In selected fermentation trials, after inoculation with* P. stipitis*, the effect of aeration was evaluated using a 0.3 vvm constant air supply. Fermentation was resumed for a further 48 hours, the entire process time amounting to 72 h. The process was controlled gravimetrically (a decrease in mass caused by the liberation of carbon dioxide). When the fermentation was complete, samples were collected to determine the ethanol, hexose, and pentose sugar concentrations.

### 2.6. Analytical Methods

The sugar beet pulp was analyzed following methods recommended for the sugar industry [22]. Solid substance was measured in a Radwag WPS-30S weighing dryer. Total nitrogen was determined using the Kjeldahl method. Reducing sugars and total sugars (after inversion with hydrochloric acid) were determined according to the Miller method [23], in g of invert sugar per kg of thick juice. The concentration of saccharose was calculated as the difference between the quantities of total sugars and reducing sugars (with a conversion coefficient of 0.95). Cellulose content was determined according to the Kürschner-Hoffer method [24], hemicellulose content using the Ernakow method [25], and lignin content following the method recommended by the National Renewable Energy Laboratory (NREL) [26]. The pH was also measured, using a digital pH meter.

The contents of glucose (GLC), fructose (FRU), galactose (GAL), xylose (XYL), arabinose (ARA), rhamnose (RHA), saccharose (SAC), cellobiose (CEL), raffinose (RAF), and galacturonic acid (GalA) in the media were determined before and after fermentation. The concentrations of ethanol in the media and in postfermentation effluents were determined using HPLC (Agilent 1260 Infinity, USA) on Hi-Plex Ca column (7.7 × 300 mm, 8 *μ*m) (Agilent Technologies, USA) equipped with a refractive index detector (RID) at 55°C. Column temperature was maintained at 80°C. HPLC grade water was used as a mobile phase at a flow rate of 0.6 mL/min with a sample volume of 20 *μ*L. Prior to analysis, samples of the worts were mixed with ZnSO_4_ to final concentrations of 10% to induce protein precipitation. The solid debris was removed by centrifugation at 4,000 rpm for 20 min. Prior to analysis, all samples were filtered through 0.45 *μ*m PES (polyethersulfone) membranes.

### 2.7. Evaluation of Hydrolysis and Fermentation

Hydrolysis yield (HY) was calculated according to the following formula:

(1) $$\begin{array}{c} HY=\frac{C*0.9}{RS+SAC+RAF+P}*100\%, \end{array}$$

where *C* is the reducing pentose and hexose sugars concentration after hydrolysis [g/L]; RS are the reducing sugars in sugar beet pulp before hydrolysis [g/L]; SAC and RAF, respectively, are saccharose and raffinose contents [g/L]; *P* is polysaccharide (cellulose and hemicellulose) content [g/L]; and 0.9 is the conversion coefficient from polysaccharide (cellulose and hemicellulose) to pentose and hexose sugars (i.e., the molecular weight ratio of polysaccharide to hexose and pentose sugars).

The total sugar intake (percentage consumption of total sugars during fermentation) was calculated as the ratio of sugars used to their content in the wort prior to fermentation, expressed as a percentage.

Fermentation efficiency (FE) was calculated for fermentable sugars (using a stoichiometric equation) and expressed as a percentage of the theoretical yield, according to the following formula:

(2) $$\begin{array}{c} FE=\frac{E}{FS*0.51}*100\%, \end{array}$$

where *E* is ethanol concentration in the fermented medium [g/L]; FS are fermentable sugars (glucose, fructose, galactose, and xylose); 0.511 is the constant which represents the theoretical yield of ethanol from glucose and xylose.

Ethanol yield was expressed as the amount of absolute ethanol (*A*_100_) obtained from 100 kg of wet sugar beet pulp.

### 2.8. Statistical Analysis

All samples were prepared and analyzed in triplicate. Statistical calculations were performed using STATISTICA 9.0 software (StatSoft, USA). The results were evaluated using one-way analysis of variance (ANOVA) and two-way ANOVA with a significance level of 0.05. Where statistical differences were found (*p* < 0.05), post hoc analysis was conducted using Tukey's range test (with a significance level of 0.05) to determine which specific means were different.

## 3. Results and Discussion

### 3.1. Chemical Composition of Sugar Beet Pulp

The chemical composition of the sugar beet pulp used in this study was typical for that of sugar beet byproducts of processing (see Table 1).

The high content of carbohydrates, in particular nonstarch polysaccharides such as cellulose and hemicellulose, and low content of lignin are advantages from the technological point of view, enabling high yields of fermentable sugars (including glucose and xylose). Sugar beet pulp feedstock therefore has great potential for use in “second-generation” biofuel (ethanol) production. However, efficient hydrolysis and fermentation depend on the type of pretreatment, the conditions of enzymatic hydrolysis, and the microorganisms used for the fermentation of released hexose and pentose sugars.

Our results are similar to others reported in the literature [27, 28]. Any differences in the chemical composition of sugar beet pulp can be related to the varieties of sugar beet processed in sugar factories, to different conditions of sugar beet cultivation, and to the technologies used for processing.

### 3.2. Effect of Pretreatment Type and Enzyme Preparation Dosage on the Release of Fermentable Sugars

Different types of pretreatment and various dosages of the cellulase and hemicellulase preparations Viscozyme and Ultraflo Max (Novozymes) were investigated to determine their effects on the release of fermentable sugars. Thermochemical pretreatment is recommended to remove most of the lignin and facilitate the action of cellulases and hemicellulases on cellulose and hemicellulose, so that the microorganisms can use the liberated monosaccharides as a carbon source [7, 8].

Due to the fact that the tested feedstock contained a relatively low lignin content, the first batch of experiments was carried out without pretreatment. A mixture of SBP and water in a ratio providing a medium containing approx. 12% d.m. was digested using different doses of the enzyme preparations Viscozyme and Ultraflo Max, in a range from 0.01 to 0.07 mL/g d.m. The samples were then incubated at 37 ± 1°C for 72 h, but without yeast to determine the degree of polysaccharide hydrolysis. The goal of this stage of the study was to determine the amounts of sugars that could potentially be released under fermentation conditions. Due to the fact that the samples were not inoculated with yeast and there was no fermentation, the worts were supplemented with the antibiotics penicillin G sodium salt (100 000 U/L wort) and streptomycin sulfate salt (0.1 g/L wort) to protect the process from bacterial contamination and prevent microbial infections.

**Figure 1:**
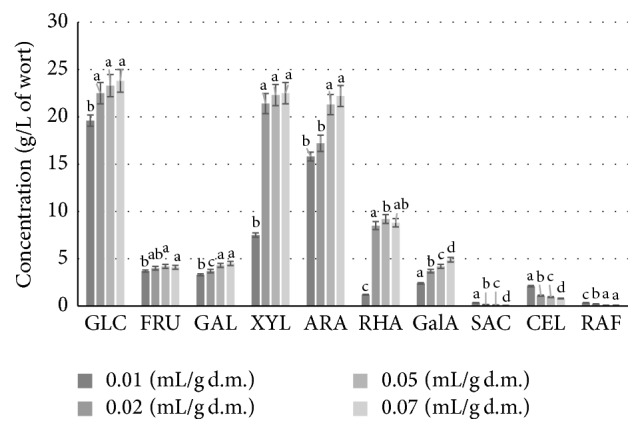
Qualitative and quantitative composition of carbohydrates in sugar beet pulp hydrolysate obtained after digestion of the feedstock (without pretreatment) with different dosage of Viscozyme and Ultraflo Max enzyme preparations. ^a–c^Mean values for each sugar content with different letters are significantly different (*p* < 0.05, one-way ANOVA).

As shown in Figure 1, enzymatic hydrolysis of SBP in water with Viscozyme and Ultraflo Max preparations (each at a dose of 0.01 mL/g d.m.) resulted in the release of the following amounts of sugars (per liter): 19.6 g glucose, 3.3 g galactose, 3.7 g fructose, 7.5 g xylose, 15.8 g arabinose, 1.2 g rhamnose, and 2.4 g galacturonic acid. Saccharose (0.35 g/L), cellobiose (2.1 g/L), and raffinose (0.35 g/L) were also found. According to the literature [3, 29], these carbohydrates can be found in sugar beet roots, explaining their presence in the SBP. Rhamnose is bound to galacturonic acid by *α*-1-4-glycosidic bonds, which form long, “smooth” regions, and *α*-1-2-glycosidic bonds, which create branched regions in the chains that build pectins [30]. The remaining rhamnose is linked by *α*-1-5-glycosidic bonds to arabinose chains [28].

Increasing the dose of enzymatic preparations to 0.02 mL/g d.m. caused a relatively small rise (approx. 15%) in glucose concentration, to 22.5 g/L (*p* > 0.05), and significant increases in the concentration of xylose and rhamnose, to 21.4 g/L and 8.5 g/L, respectively (*p* < 0.05). Further increasing the enzyme preparation dosage to 0.05 mL/g d.m. of sugar beet pulp resulted in increased galactose, arabinose, and rhamnose contents (*p* < 0.05). However, a further increase to 0.07 mL/g d.m. did not significantly improve the efficiency with which sugars were released (*p* > 0.05).

The highest efficiency of hydrolysis (72 ± 3%) was observed in batches where the enzymatic preparations were applied in doses of 0.02 mL/g d.m. (*p* < 0.05). Higher doses of these preparations did not result in the release of greater amounts of glucose and xylose (*p* > 0.05). A significant increase occurred only in the case of arabinose (*p* < 0.05). The inhibition of cellulase and hemicellulase activities observed may have been caused by the rising concentration of sugars, as well as by the limited access of these enzymes to substrates contained in the feedstock.

**Figure 2:**
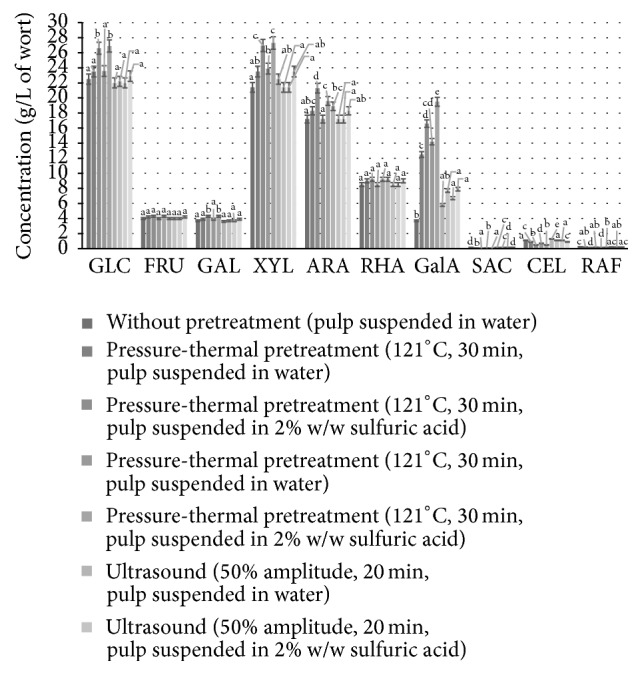
Qualitative and quantitative composition of carbohydrates in sugar beet pulp hydrolysate obtained after digestion of the sugar beet pulp with Viscozyme and Ultraflo Max enzyme preparations (each at a dose of 0.02 mL/g d.m.), preceded by different pretreatments. ^a–e^Mean values for each sugar content with different letters are significantly different (*p* < 0.05, one-way ANOVA).

The next stage of the investigation focused on whether pretreatment of the ground beet pulp improved the release of fermentable sugars. The following forms of pretreatment were considered: suspension in water or in 2% w/w sulfuric acid solution (ratio of the pulp to water or acid approx. 12% dry matter in the medium); pretreatment by autoclaving at 121°C for 30 or 60 min; ultrasound action (amplitude 50 or 100%, 20 min). Enzymatic hydrolysis was then performed with cellulase and hemicellulase preparations, each at doses of 0.02 mL/g d.m. The results are presented in Figure 2.

**Figure 3:**
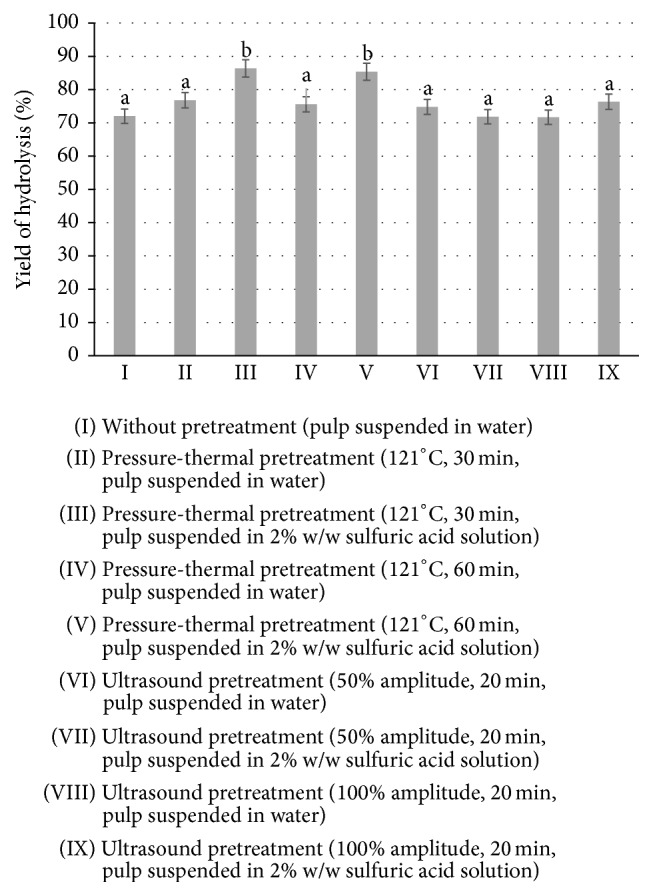
Yield of hydrolysis of polysaccharides in the tested SBP after pretreatment and enzymatic hydrolysis with Viscozyme and Ultraflo Max, each at a dose of 0.02 mL/g d.m., under fermentation conditions (37 ± 1°C, 72 h). ^a-b^Mean values with different letters are significantly different (*p* < 0.05, one-way ANOVA).

The highest release rate of fermentable sugars was observed after 30 minutes at 121°C from sugar beet pulp suspended in 2% w/w sulfuric acid solution. Most significantly, the concentration of glucose increased by 18% (from 22.5 to 26.6 g/L), and that of xylose increased by 26% (from 21.4 to 26.96 g/L) (*p* < 0.05). Conversely, the concentrations of cellobiose, raffinose, and saccharose dropped to 0.05–0.13 g/L of wort. The highest yield of polysaccharides and oligosaccharides was also observed with this variant of the experiment, reaching 86.4 ± 2.6% (*p* < 0.05). Neither increasing the time of thermal treatment to 60 minutes nor applying ultrasound treatment yielded higher concentrations of fermentable sugars (Figure 3) (*p* < 0.05). These results are in agreement with data obtained by Rezić et al. [31], which showed that pressure-thermal pretreatment had an effect on lignocellulosic substrates and favored the release of monosaccharides from cellulose and hemicellulose. Dilute acid hydrolysis treatment also caused disruption to the polymetric structure of the sugar beet pulp [32].

### 3.3. Results of Simultaneous Saccharification and Fermentation of Sugar Beet Pulp-Based Worts

Earlier research had demonstrated that conducting separate enzymatic hydrolysis of sugar beet pulp at elevated temperatures (approx. 48–50°C) for 48 hours increased the cost of the process and the risk of microbial infection, thereby lowering fermentation performance (data not shown). Therefore, this study focused on selecting optimal conditions for simultaneous saccharification and fermentation (SSF) of sugar beet pulp-based worts. SSF eliminates the time needed for separate hydrolysis of the polysaccharides present in sugar beet pulp. Consequently, it reduces the total time required for the process, from preparation of the feedstock to obtaining the final product. Based on our prior research, Ethanol Red distillery yeast was selected to ferment the hexose sugars. This strain exhibits tolerance to variable conditions (pH 3.5–6.0, temperature up to 40°C), which is especially important in SSF processes [33]. Also, in previous studies, the most efficient yeast for the fermentation of pentoses (e.g., xylose) had been found to be* Pichia stipitis* NCYC 1541 (the National Collection of Yeast Cultures, UK). Sequential inoculation was used, initiated with the dry distillery yeast (1 g d.m./L) followed by inoculation after 24 h with* Pichia stipitis* (0.5 d.m. g/L).

To investigate whether partial saccharification of the polysaccharides (so-called “enzymatic activation”) had an impact on the effectiveness of the SSF process, we introduced an interval between the application of the enzymatic preparations (0.02 mL/g d.m.) and inoculation with yeast. After 6 hours of activation, the partially hydrolyzed biomass was inoculated with Ethanol Red yeast. After 24 h of fermentation,* P. stipitis* yeast inoculum was added. The results are presented in Table 2.

The lowest ethanol content (14.3 ± 0.6 g/L) was determined in the control sample, in which simultaneous saccharification and fermentation was performed without pretreatment (*p* < 0.05). When an analogous fermentation trial (pulp suspended in water, without pretreatment of SBP) was subjected to 6 h of enzymatic activation and then inoculated with yeast, the ethanol concentration after fermentation increased by 16.8% to 16.7 ± 0.5 g/L (*p* < 0.05). Fermentation efficiency rose from 54.2 ± 2.2% to 63.3 ± 1.8% of the theoretical yield.

The highest amount of ethanol (*p* < 0.05) was measured in the trial subjected to enzymatic activation (Variant I, inoculation with yeast Ethanol Red after 6 h action with Viscozyme 0.02 mL/g d.m. and Ultraflo Max 0.02 mL/g d.m. at 40°C), preceded by 30 min pressure-thermal pretreatment of the pulp suspended in 2% w/w sulfuric acid. Ethanol concentration in this wort reached 26.9 ± 1.2 g/L, while the fermentation efficiency was 86.5 ± 2.1% of the theoretical yield. Intake of hexose sugars (i.e., glucose, fructose, and galactose) was 90.3 ± 2.2%, whereas 87.2 ± 1.9% of the xylose was used.

Similar fermentation factors were observed in Variant II of the experiment, in which enzymatic activation was performed for 6 h with lower doses of the preparations (0.015 mL/g d.m. Viscozyme and 0.015 mL/g d.m. Ultraflo Max), at higher temperatures (48–50°C) than in Variant I, but with analogous pretreatment (Table 2). Thermochemical pretreatment followed by activation using enzymatic preparations in doses between 0.015 and 0.02 mL/g d.m. of SBP and inoculation with yeast led to increased ethanol concentrations (*p* < 0.05) and more dynamic fermentation. As a consequence, the fermentation process was completed within 60 hours with no loss of CO_2_.

Prolonging the period of pressure-thermal treatment from 30 to 60 min did not significantly improve the results for fermentation of SBP suspended in 2% w/w sulfuric acid (*p* > 0.05). Pretreatment with ultrasound before enzymatic hydrolysis did not improve the efficiency of fermentable sugar release or fermentation factors (Table 2) (*p* > 0.05). There was a statistically significant reduction in fermentation performance (i.e., ethanol concentration and process efficiency) with all fermentation batches of worts prepared from SBP pretreated with ultrasound waves, compared with those for analogous fermentation trials in which pressure-thermal pretreatment was applied (*p* < 0.05).

The most favorable variant was pressure-thermal pretreatment of SBP suspended in 2% w/w sulfuric acid, followed by simultaneous saccharification and fermentation of wort subjected to enzymatic activation. This variant enabled high utilization of fermentable sugars and maximal ethanol yield under the experimental conditions described above.

The concentrations of arabinose, rhamnose, and galacturonic acid reduced by similar proportions in all the fermentation trials, between 3 and 5% (data not shown), and did not differ statistically compared to the worts before fermentation. This indicates that the yeast strains used in our study did not assimilate these compounds. Patelski et al. [34], who investigated the bioconversion of sugar beet pulp into single-cell protein (SCP) using* Saccharomyces cerevisiae* and* Pichia stipitis* yeast strains, observed that glucose, fructose, and galactose were assimilated by all the tested strains.* S. cerevisiae* was not able to utilize xylose, arabinose, rhamnose, or galacturonic acid, while the* P. stipitis* strain utilized only approx. 15% of the arabinose and 40% of the rhamnose. It is notable that the* P. stipitis* yeast strain used by Patelski et al. was not found to utilize xylose as a carbon source for biomass production.

Recent research has investigated the possibility of improving the fermentation of arabinose by using recombinant yeast strains [35]. Bettiga et al. [36] investigated pentose fermentation using recombinant* S. cerevisiae* strains. Under anaerobic conditions, a yeast strain containing a complete L-arabinose pathway fermented L-arabinose in the presence of glucose. The authors observed minor coconsumption of L-arabinose in the presence of glucose, but after glucose depletion the consumption rate was higher and subsequently pentose fermentation was observed.

**Figure 4:**
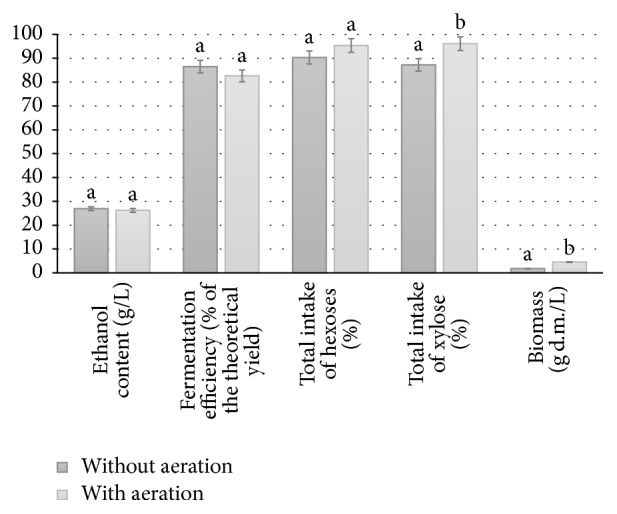
Effect of aeration on efficiency of simultaneous saccharification and fermentation of sugar beet pulp (fermentation Variant I with enzymatic activation: inoculation with yeast Ethanol Red after 6 h of enzymatic action (Viscozyme 0.02 mL/g d.m.; Ultraflo Max 0.02 mL/g d.m., 40°C), preceded by 30 min pressure-thermal pretreatment of SBP suspended in 2% w/w sulfuric acid solution). ^a-b^Mean values for each index with different letters are significantly different (*p* < 0.05, one-way ANOVA).

According to the literature, the ability of* Pichia stipitis* yeast to metabolize xylose is dependent on culture oxygenation [16, 17]. It has been reported that ethanol yield can be significantly increased when* q*O2 (the specific oxygen uptake rate) is adjusted to the optimum oxygen level for the type of sugar consumed [37]. According to our results, aeration did not improve the effectiveness of SBP fermentation. However, statistically significant increases in the consumption of hexose sugars and xylose were observed (*p* < 0.05), and as a consequence we noted an approximately threefold increase in the yeast biomass (*p* < 0.05) (see Figure 4). The recovery of so-called postfermentation yeast, which is a valuable source of protein (over 50% d.m.), can significantly improve the economic viability of the entire process.

Gutiérrez-Rivera et al. [38] observed that, in coculture experiments (simultaneous inoculation of* S. cerevisiae* ITV-01 and* P. stipitis* NRRL Y-7124), ethanol production did not depend on the level of aeration, while ethanol productivity was higher in cocultures with an air supply compared to those without (1.26 and 0.38 g L^−1^ h^−1^, resp.). Moreover, ethanol productivity from aerated cocultures was greater than with* P. stipitis* NRRL Y-7124 alone under the same conditions (0.24 and 1.26 g L^−1^ h^−1^, resp.). Yet, whereas with* P. stipitis* NRRL Y-7124 100% of the xylose was used, with aerated cocultures xylose uptake was incomplete (79.6%). Gutiérrez-Rivera et al. conclude that it is probable that* S. cerevisiae* ITV-01 and* P. stipitis* NRRL Y-7124 adversely affect each other. This may be due to the limited oxygen available for* P. stipites*, as a result of oxygen utilization by* S. cerevisiae*.

On the basis of our fermentation results, we calculated the quantity of ethanol obtained from 100 kg of sugar beet pulp-based wort. Our study shows that 6.8 ± 0.3 kg absolute ethanol can be produced from 100 kg of wet sugar beet pulp (approx. 23% d.m.) under the most favorable conditions, as established in our experiments (see Table 3).

## 4. Conclusions

The results of our study suggest that sugar beet pulp, an inexpensive byproduct of sugar beet processing, could provide an alternative feedstock for second-generation ethanol production. To ensure the effectiveness of simultaneous saccharification and fermentation, enzymatic hydrolysis should be preceded by pressure-thermal pretreatment (121°C, 30 min) of sugar beet pulp suspended in 2% w/w sulphuric acid in a ratio providing 12% dry matter. A 6 h interval for enzymatic activation between the start of digestion with enzyme preparations and inoculation with the yeast strain may improve fermentation performance. Fermentation of progressively released fermentable hexose (glucose, fructose, and galactose) and pentose (xylose) sugars by two yeast strains applied sequentially, Ethanol Red (*S. cerevisiae*) and* Pichia stipitis* NCYC 1541, yielded 6.8 ± 0.3 kg absolute ethanol from 100 kg of wet sugar beet pulp (approx. 23% dry matter). To increase the intake of released monomer sugars, strains of yeast able to ferment rhamnose, arabinose, and galacturonic acid should also be added.

## Figures and Tables

**Table 1:** Chemical composition of raw material.

Physicochemical parameters	Sugar beet pulp
Dry mass (g/kg)	229.3 ± 11.5
pH	5.8 ± 0.2
Reducing sugars as invert sugar (g/kg d.m.)	9.8 ± 0.3
Saccharose (g/kg d.m.)	144.8 ± 12.5
Raffinose (g/kg d.m.)	2.4 ± 0.3
Cellulose (g/kg d.m.)	336.8 ± 15.2
Hemicellulose (g/kg d.m.)	405.5 ± 27.2
Lignin (g/kg d.m.)	1.4 ± 0.2
Protein (*N* × 6.25) (g/kg d.m.)	11.5 ± 0.25

Results expressed as mean values ± SE (*n* = 3).

**Table 2:** Effect of simultaneous saccharification and fermentation conditions on sugar beet pulp-based wort fermentation factors and intake of sugars.

Fermentation trial	Parameters	Pretreatment								
		Without pretreatment, pulp suspended in water	Pressure-thermal pretreatment				Ultrasound pretreatment			
			30 min		60 min		50% amplitude, 20 min		100% amplitude, 20 min	
			Pulp suspended in water	Pulp suspended in 2% w/w sulfuric acid solution	Pulp suspended in water	Pulp suspended in 2% w/w sulfuric acid solution	Pulp suspended in water	Pulp suspended in 2% w/w sulfuric acid solution	Pulp suspended in water	Pulp suspended in 2% w/w sulfuric acid solution
Initial fermentable sugar content as sum of glucose, fructose, galactose, saccharose, and xylose [g/L]		51.75 ± 1.55^a^	55.18 ± 1.65^a^	62.21 ± 1.86^b^	55.45 ± 1.65^a^	62.85 ± 1.90^b^	52.23 ± 1.40^a^	51.43 ± 1.54^a^	51.27 ± 1.54^a^	54.67 ± 1.64^a^

Without enzymatic activation^*∗*^	Ethanol content (g/L)	14.3 ± 0.6^a^	19.9 ± 0.7^efgh^	22.5 ± 1.1^hij^	20.5 ± 0.8^fghi^	22.7 ± 0.9^ij^	15.8 ± 0.4^ab^	16.3 ± 0.5^abc^	16.2 ± 0.5^abc^	17.5 ± 0.7^bcde^
	Fermentation efficiency (% of the theoretical yield)	54.2 ± 2.2^a^	70.7 ± 2.3^cdefgh^	70.9 ± 3.5^defgh^	72.5 ± 2.8^fghi^	80.3 ± 3.1^ij^	59.4 ± 1.5^ab^	62.2 ± 1.9^abcd^	62.1 ± 1.9^abc^	62.7 ± 2.5^abcde^
	Intake of hexoses (%)	85.1 ± 2.4^abcdef^	83.2 ± 2.5^abc^	83.8 ± 2.2^abcd^	84.2 ± 2.6^abcde^	83.8 ± 2.5^abcd^	82.1 ± 2.4^a^	82.8 ± 2.5^ab^	85.2 ± 2.5^abcdef^	85.1 ± 2.4^abcdef^
	Intake of xylose (%)	65.3 ± 1.5^a^	75.2 ± 1.8^cdef^	77.2 ± 1.5^defgh^	76.2 ± 1.4^cdefg^	77.5 ± 1.6^defgh^	67.3 ± 1.5^ab^	74.5 ± 1.5^cd^	76.2 ± 1.5^cdefg^	71.3 ± 1.5^bc^

Enzymatic activation, Variant I^*∗∗*^	Ethanol content (g/L)	16.7 ± 0.5^abcd^	22.5 ± 0.7^hij^	26.9 ± 1.2^k^	23.6 ± 0.9^j^	27.1 ± 1.2^k^	17.5 ± 0.6^bcde^	18.3 ± 0.7^bcdef^	18.1 ± 0.6^bcdef^	18.7 ± 0.8^cdef^
	Fermentation efficiency (% of the theoretical yield)	63.3 ± 1.8^bcde^	80.1 ± 2.3^ij^	86.5 ± 2.1^j^	83.4 ± 3.2^j^	84.4 ± 3.7^j^	65.8 ± 2.2^bcdefg^	69.8 ± 2.7^cdefgh^	69.3 ± 2.3^cdefg^	67.0 ± 2.9^bcdefg^
	Intake of hexoses (%)	91.6 ± 2.5^efgh^	88.2 ± 2.2^abcdefgh^	90.3 ± 2.4^bcdefgh^	90.5 ± 2.5^cdefgh^	95.5 ± 2.6^h^	89.2 ± 2.5^abcdefgh^	88.6 ± 2.2^abcdefgh^	88.2 ± 2.2^abcdefgh^	87.5 ± 2.5^abcdefg^
	Intake of xylose (%)	74.5 ± 2.5^cd^	86.5 ± 2.6^jkl^	88.2 ± 1.9^kl^	87.5 ± 1.8^kl^	89.5 ± 1.5^l^	83.5 ± 1.6^ijk^	80.5 ± 1.2^efghi^	82.5 ± 1.5^hijk^	81.5 ± 1.4^ghij^

Enzymatic activation, Variant II^*∗∗*^	Ethanol content (g/L)	16.9 ± 0.5^abcd^	22.1 ± 0.8^ghij^	26.6 ± 1.5^k^	22.7 ± 0.8^ij^	26.9 ± 1.5^k^	18.1 ± 0.6^bcdef^	18.6 ± 0.8^cdef^	19.2 ± 0.7^def^	19.6 ± 0.7^efg^
	Fermentation efficiency (% of the theoretical yield)	64.3 ± 1.6^bcdef^	78.5 ± 2.8^hij^	83.8 ± 4.7^j^	80.3 ± 2.7^ij^	83.8 ± 4.2^j^	68.0 ± 2.3^bcdefg^	71.0 ± 2.8^efgh^	73.6 ± 2.6^ghi^	70.3 ± 2.5^cdefgh^
	Intake of hexoses (%)	91.3 ± 2.2^defgh^	88.2 ± 2.6^abcdefgh^	92.0 ± 2.5^fgh^	89.5 ± 1.8^abcdefgh^	93.0 ± 1.5^gh^	88.3 ± 2.2^abcdefgh^	88.5 ± 2.2^abcdefg^	86.2 ± 2.5^abcdefg^	88.2 ± 2.4^abcdefgh^
	Intake of xylose (%)	74.9 ± 1.9^cde^	83.3 ± 2.2^ijk^	88.1 ± 2.2^kl^	85.2 ± 2.2^ijkl^	89.5 ± 2.2^l^	74.9 ± 1.9^cde^	80.8 ± 1.5^fghi^	80.3 ± 2.0^efghi^	80.5 ± 1.5^efghi^

Results expressed as mean values ± SE (*n* = 3); ^a-b^mean values for initial fermentable sugar content with different letters are significantly different (*p* < 0.05, one-way ANOVA); ^a–l^mean values for ethanol content, fermentation efficiency, intake of hexoses, and intake of xylose with different letters are significantly different (*p* < 0.05, two-way ANOVA).

^*∗*^Without enzymatic activation: inoculation with yeast Ethanol Red immediately after application of enzyme preparations.

^*∗∗*^Enzymatic activation, Variant I: inoculation with yeast Ethanol Red after 6 h of enzymatic preparations action (Viscozyme 0.02 mL/g d.m.; Ultraflo Max 0.02 mL/g d.m., 40°C). Enzymatic activation, Variant II: inoculation with yeast Ethanol Red after 6 h of enzymatic preparations action (Viscozyme 0.015 mL/g d.m.; Ultraflo Max 0.015 mL/g d.m., 48–50°C).

**Table 3:** Ethanol yield (kg absolute ethanol) from 100 kg of wet (ca. 23% d.m.) sugar beet pulp.

Fermentation trial	Pretreatment								
	Without pretreatment, pulp suspended in water	Pressure-thermal pretreatment				Ultrasound pretreatment			
		30 min		60 min		50% amplitude, 20 min		100% amplitude, 20 min	
		Pulp suspended in water	Pulp suspended in 2% w/w sulfuric acid solution	Pulp suspended in water	Pulp suspended in 2% w/w sulfuric acid solution	Pulp suspended in water	Pulp suspended in 2% w/w sulfuric acid solution	Pulp suspended in water	Pulp suspended in 2% w/w sulfuric acid solution
Without enzymatic activation^*∗*^	3.6 ± 0.2^a^	5.0 ± 0.2^efgi^	5.6 ± 0.3^ijk^	5.1 ± 0.2^ghij^	5.7 ± 0.2^jk^	3.9 ± 0.1^ab^	4.1 ± 0.2^abc^	4.1 ± 0.1^abc^	4.4 ± 0.2^bcdef^

Enzymatic activation, Variant I^*∗∗*^	4.2 ± 0.1^abcd^	5.6 ± 0.2^ijk^	6.7 ± 0.3^h^	5.9 ± 0.2^k^	6.8 ± 0.3^l^	4.4 ± 0.2^bcdef^	4.6 ± 0.2^cdefg^	4.5 ± 0.2^bcdefg^	4.7 ± 0.2^cdefg^

Enzymatic activation, Variant II^*∗∗*^	4.2 ± 0.1^abcd^	5.5 ± 0.2^hijk^	6.7 ± 0.4^jk^	5.7 ± 0.2^jk^	6.7 ± 0.4^bcdefg^	4.5 ± 0.2^bcdefg^	4.7 ± 0.2^cdefg^	4.8 ± 0.2^defg^	4.9 ± 0.2^efg^

Enzymatic activation, Variant I & aeration	4.3 ± 0.1^bcde^	5.7 ± 0.2^bcde^	6.6 ± 0.3^jk^	5.6 ± 0.2^ijk^	6.6 ± 0.3^l^	4.7 ± 0.1^cdefg^	4.9 ± 0.2^efg^	4.9 ± 0.2^efg^	5.0 ± 0.2^fghi^

Results expressed as mean values ± SE (*n* = 3); ^a–l^mean values with different letters are significantly different (*p* < 0.05, two-way ANOVA).

## Abbreviations

SSF: Simultaneous saccharification and fermentation
SBP: Sugar beet pulp
vvm: Aeration rate = gas volume flow per unit of liquid volume per minute (volume per volume per minute)
d.m.: Dry matter
GLC: Glucose
GAL: Galactose
FRU: Fructose
XYL: Xylose
ARA: Arabinose
RHA: Rhamnose
GalA: Galacturonic acid
SAC: Saccharose
CEL: Cellobiose
RAF: Raffinose.
